# rTMS neuromodulation improves electrocortical functional measures of information processing and behavioral responses in autism

**DOI:** 10.3389/fnsys.2014.00134

**Published:** 2014-08-06

**Authors:** Estate M. Sokhadze, Ayman S. El-Baz, Lonnie L. Sears, Ioan Opris, Manuel F. Casanova

**Affiliations:** ^1^Department of Psychiatry and Behavioral Sciences, University of LouisvilleLouisville, KY, USA; ^2^Department of Bioengineering, University of LouisvilleLouisville, KY, USA; ^3^Department of Pediatrics, University of LouisvilleLouisville, KY, USA; ^4^Department of Physiology and Pharmacology, Wake Forest UniversityWinston-Salem, NC, USA

**Keywords:** TMS, autism, ERP, motor response time, behavioral performance

## Abstract

**Objectives:** Reports in autism spectrum disorders (ASD) of a minicolumnopathy with consequent deficits of lateral inhibition help explain observed behavioral and executive dysfunctions. We propose that neuromodulation based on low frequency repetitive Transcranial Magnetic Stimulation (rTMS) will enhance lateral inhibition through activation of inhibitory double bouquet interneurons and will be accompanied by improvements in the prefrontal executive functions. In addition we proposed that rTMS will improve cortical excitation/inhibition ratio and result in changes manifested in event-related potential (ERP) recorded during cognitive tests.

**Materials and Methods:** Along with traditional clinical behavioral evaluations the current study used ERPs in a visual oddball task with illusory figures. We compared clinical, behavioral and electrocortical outcomes in two groups of children with autism (TMS, wait-list group). We predicted that 18 session long course in autistic patients will have better behavioral and ERP outcomes as compared to age- and IQ-matched WTL group. We used 18 sessions of 1 Hz rTMS applied over the dorso-lateral prefrontal cortex in 27 individuals with ASD diagnosis. The WTL group was comprised of 27 age-matched subjects with ASD tested twice. Both TMS and WTL groups were assessed at the baseline and after completion of 18 weekly sessions of rTMS (or wait period) using clinical behavioral questionnaires and during performance on visual oddball task with Kanizsa illusory figures.

**Results:** Post-TMS evaluations showed decreased irritability and hyperactivity on the Aberrant Behavior Checklist (ABC), and decreased stereotypic behaviors on the Repetitive Behavior Scale (RBS-R). Following rTMS course we found decreased amplitude and prolonged latency in the frontal and fronto-central N100, N200 and P300 (P3a) ERPs to non-targets in active TMS treatment group. TMS resulted in increase of P2d (P2a to targets minus P2a to non-targets) amplitude. These ERP changes along with increased centro-parietal P100 and P300 (P3b) to targets are indicative of more efficient processing of information post-TMS treatment. Another important finding was decrease of the latency and increase of negativity of error-related negativity (ERN) during commission errors that may reflect improvement in error monitoring and correction function. Enhanced information processing was also manifested in lower error rate. In addition we calculated normative post-error treaction time (RT) slowing response in both groups and found that rTMS treatment was accompanied by post-error RT slowing and higher accuracy of responses, whereas the WTL group kept on showing typical for ASD post-error RT speeding and higher commission and omission error rates.

**Conclusion:** Results from our study indicate that rTMS improves executive functioning in ASD as evidenced by normalization of ERP responses and behavioral reactions (RT, accuracy) during executive function test, and also by improvements in clinical evaluations.

## Introduction

Autism Spectrum Disorders (ASD) are featured by severe deficits in social communication, social interaction, and restricted, repetitive patterns of behaviors, interests and activities (APA, 2013). Additionally, autistic individuals usually present excessive reactions to the sensory environment such as aversive reactions to visual, auditory, and tactile stimuli. These perception and sensory reactivity abnormalities are found in majority of subjects with ASD affecting their ability to effectively process information (Gomes et al., 2008). In a series of electrophysiological studies conducted by our group we explored specifics of event-related potential (i.e., ERP) reflecting information processing during performance on reaction time (RT) tasks in children with ASD (Sokhadze et al., 2009a, 2012b, 2013a; Baruth et al., 2010c; Casanova et al., 2012) Our studies were aimed to explore the manifestations of the impaired functional connectivity, excessive cortical excitation/inhibition ratio, and deficient executive functioning in ASD by analyzing behavioral performance on attention tasks with dense-array ERP recording. Analysis of ERP components is one of the most informative dynamic methods of investigation and monitoring of information processing stages in the human brain due to the high temporal resolution of this technique. Amplitude and latency of ERP waves at selected topographies reflect both early sensory perception processes and higher-level processing including attention, cortical inhibition, memory update, as well as other cognitive activity processes (Polich, 2007). ERPs provide both a method of studying chronometry of information processing stages and a tool by which to assess the neurobiology of cognitive dysfunctions present in this neurodevelopmental disorder. ERP is a very useful technique to characterize time course and amplitude of cortical responses to stimulation (Jeste and Nelson, 2009). Generally, early exogenous ERPs are believed to reflect sensory processing of a stimulus attributes (Coles and Rugg, 1995; Herrmann and Knight, 2001; Eichele et al., 2005; Folstein et al., 2008), whereas late endogenous ERPs are thought to reflect higher level cognitive processes such as attention, memory trace update, perceptual closure, etc. (Pritchard, 1981; Picton, 1992; Polich, 2003, 2007).

One of our first studies investigated ERPs that index selective attention processes in a visual novelty oddball task in children with autism and an age-matched group of typically developing children (Sokhadze et al., 2010a). The ASD group had excessive magnitude to task-irrelevant visual cues as compared to typically developing children and evidenced a lack of visual target discrimination. In a follow-up investigation we found augmented early cortical responses to novel distracters along with lower accuracy of motor response (MR) in a three-stimuli oddball task with illusory Kanizsa figures (Sokhadze et al., 2013a). We concluded that cortical responses to visual stimulation in autism might be indiscriminative during visual tasks negatively affecting selective attention. Large magnitude of electrocortical activity in response to sensory stimulation may be due to an increased ratio between excitation and inhibition in the cortex of individuals with autism (Casanova et al., 2002a,b; Rubenstein and Merzenich, 2003; Casanova, 2005, 2007). Impaired habituation and normative adaptation to repeated stimuli can be considered as an inhibitory deficit manifested in typical symptoms of autism such as stereotypy, sensory hypersensitivity, deficient social interaction skills, etc.

One of contemporary models of autism, so called “minicolumnar theory of autism” (Casanova et al., 2003, 2006a,b; Casanova, 2005, 2007) is based on neuropathological findings in our laboratory. Autism in this model is associated with cortical neurodevelopmental abnormalities. In brief, the reduced neuropil space (periphery of the minicolumn) reported in autism is the compartment where lateral inhibition sharpens the borders of minicolumns and increases their definition (Favorov and Kelly, 1994a,b; DeFelipe, 1999, 2004). The primary source of for this inhibitory effect may be derived from axon bundles of double-bouquet cells (Favorov and Kelly, 1994a). The axons of double bouquet cells arrange themselves in essentially repeatable patterns varying between 15 and 30 μm wide, depending on the cortical area examined (DeFelipe, 1999). Increases in numbers and types of inhibitory interneurons, as seen in the smaller minicolumns of autistic patients, result in greater diversity and more nuanced modulation of minicolumns. Double-bouquet cells in the peripheral neuropil space of minicolumns provide a “vertical stream of negative inhibition” (Mountcastle, 2003) surrounding the minicolumnar core. Other GABAergic cells in the minicolumn, having collateral projections extending hundreds of microns tangentially, provide lateral inhibition of surrounding minicolumns on a macrocolumnar scale.

The value of each minicolumn’s output is insulated to a greater or lesser degree from the activity of its neighbors by GABAergic inhibition in its peripheral neuropil space. This allows for gradations in amplitude of excitatory activity across a minicolumnar field. Rubenstein and Merzenich (2003) have posited that reductions in GABAergic inhibitory activity may explain some symptomatology of autism, including increased incidence of seizures and auditory-tactile hypersensitivity (see also Casanova et al., 2003, 2006a,b). Oblak et al. (2010) found decreased GABA receptors in the cingulate cortex and fusiform gyrus in autism. These results may explain some symptomatology of autism, including increased incidence of seizures and sensory (e.g., auditory, tactile) hypersensitivity (Casanova et al., 2003).

This hypothesis is consistent with findings of reduced minicolumnar peripheral neuropil space in the neocortex of autistics relative to controls (Casanova et al., 2002a,b,d). In this model, a reduction in the peripheral neuropil space would result in smaller minicolumns which would coalesce into discrete, isolated islands of coordinated excitatory activity. There are considerable consequences resulting from the significant reduction of neuropil in minicolumns in autism. Reduced surround inhibition may result in an increase in the ratio of cortical excitation to inhibition and excessive amplification of sensory responses reported by autistic individuals. Several important functions of the prefrontal cortex, for instance executive functions might be affected ability of individuals with autism focus on task-relevant targets without being distracted by task-irrelevant cues (Gray et al., 2003; Folstein et al., 2008; Matzel and Kolata, 2010). There are several reviews describing consequences of increased excitation-to-inhibition (E/I) ratio both in humans and in animal models (Rubenstein and Merzenich, 2003; Renart et al., 2010; Harris and Thiele, 2011; Pinto et al., 2013). Deficits within the inhibitory elements that surround the cell minicolumn suggest a mechanistic explanation to the I/E imbalance in autism (Casanova et al., 2003). Oscillations and synchronization of pyramidal cells in and across minicolumns are maintained by networks of inhibitory GABAergic interneurons. Local I/E interactions shape neuronal representations of sensory, motor and cognitive variables, and produce local electroencephalographic (EEG) gamma oscillations. The I/E bias caused by faulty pyramidal cell-interneuronal diads provides a receptive scenario to induced gamma frequency and ERP abnormalities in autism.

TMS offers a noninvasive method for altering excitability of the neural circuits and for inducing a functional reorganization of the cortex. We reported positive effects of repetitive transcranial magnetic stimulation (rTMS) in ASD in our pilot studies using shorter (6–12 sessions) rTMS course (Sokhadze et al., 2009b, 2010a, 2012a; Baruth et al., 2010a,b, 2011; Casanova et al., 2012). TMS-based neuromodulation exerts effects on cortical excitability (Maeda et al., 2000; Pascual-Leone et al., 2000, 2002; Frye et al., 2008; Baruth et al., 2010b; Enticott et al., 2010; Sokhadze et al., 2012a; Oberman et al., 2013). It is proposed that that low-frequency (i.e., “slow”’) rTMS (≤1 Hz) has inhibitory effects on stimulated cortex (Maeda et al., 2000), whereas high-frequency rTMS (>1 Hz, e.g., 5 Hz, 10 Hz etc.) increases excitability of stimulated cortex (Pascual-Leone et al., 1994, 2000, 2002; Daskalakis et al., 2002; Schutter, 2009; Wassermann and Zimmermann, 2012; Oberman et al., 2013). Probably the effect of low frequency rTMS are mediated through increases in the activation of inhibitory neurons (Hoffman and Cavus, 2002; Wagner et al., 2009). We propose that inhibitory cells such as basket and chandelier inter-neurons, whose projections keep no constant relation to the surface of the cortex, of the double-bouquet neurons are oriented in more geometrically exact manner and are located at the periphery of the minicolumn and therefore they are more appropriate candidate for induction by a TMS applied parallel to cortex. Low frequency rTMS in autism, may lower the cortical excitation/inhibition ratio, so called E/I ratio index.

In this study we were interested in how rTMS treatment affects specific ERP components known to index processes in sensory cortex, association cortical areas, and areas related to higher level cognitive activity. As it was mentioned above, the exogenous ERPs reflect early-stage, modality-specific, while endogenous ones reflect modality non-specific associative higher order processing of stimuli within the context of the task (Näätänen et al., 1978; Luck et al., 1990; Coles and Rugg, 1995; Hillyard and Annlo-Vento, 1998). Posterior visual P100 are generated within the fusiform gyrus with contribution from parieto-occipital and occipital cortices (Yamazaki et al., 2000). Frontal N100 ERP wave occurs within a similar time window and probably originates from more anterior frontal dipole generators (Clark et al., 1994).

The fronto-central P300 (so called P3a) reflects frontal lobe activity (Friedman et al., 1993) and in a visual oddball task with distracters is interpreted as an attentional “orienting”, whereas centro-parietal and parietal P300 (P3b) is believed to reflect sustain attention and other higher level processes. This cognitive ERP component has multiple dipole sources (Townsend et al., 2001).

Negative N200 component is recorded in visual tasks over centro-parietal cortex around 200–300 ms post-stimulus (Näätänen et al., 1978, 1993) and reflects processes of stimulus categorization, perceptual closure and attention focusing signaling that a perceptual representation has been formed (Potts et al., 2004). A frontal positivity with a peak within (P2a, 180–320 ms post-stimulus) over inferior prefrontal recording sites is selectively responsive to the evaluation of the task relevance of presented stimuli, and originates from the orbito-frontal cortex (Potts et al., 1996, 1998, 2008). This frontal component may index task-relevant features of the stimulus (Kenemans et al., 1993). The fronto-central N200 according to some researchers (West, 2003; Donkers and van Boxtel, 2004; West et al., 2004) is thought to originate from the anterior cingulate cortex (ACC) and prefrontal sources and may reflect processes related to potential response conflict detection in a RT tasks and/or cortical inhibition of inappropriate MR. All ERP components have certain variability, but specific ERP measures selected for this study (frontal N100, N200 and P300, and parietal P100, N200 and P300) are less affected by variability in visual tasks and are relevant to the study goal.

We proposed that after 18 sessions of 1 Hz rTMS—administered to the dorso-lateral prefrontal cortices (DLPFC) participants with autism would demonstrate normalization of electrocortical indices of attention both at the early (P100, N100, 100–200 ms post-stimulus) and the late (i.e., P200, N200, P300, 200–600 ms) stages of sensory and cognitive processing and show improvements in MT accuracy. Mainly, we expected lower magnitude and longer latencies to visual targets (i.e., better stimulus discrimination), and attenuated reactivity to non-target illusory figures and other non-target cues. Other anticipated improvements were expected to be found in outcomes of social and behavioral functioning questionnaire and surveys. The hypothesis in this study proposed that low-frequency rTMS (i.e., inhibitory) would exert its effects through increased cortical inhibitory tone (i.e., lower E/I ratio) in the DLPFC with subsequent improvement in performance in the visual attention task. In addition we expected improvements in clinical social and behavioral evaluation outcomes.

## Methods

Participants with ASD (age range 9–21 years) were recruited through the University of Louisville Weisskopf Child Evaluation Center (WCEC). Diagnosis was made according to the Diagnostic and Statistical Manual of Mental Disorders (DSM-IV-TR) (APA, 2000) and further ascertained with the Autism Diagnostic Interview—Revised (ADI-R; Le Couteur et al., 2003). They also had a medical evaluation by a developmental pediatrician. All subjects had normal hearing based on past hearing screens. Participants with a history of seizure disorder, significant hearing or visual impairment, a brain abnormality conclusive from imaging studies or an identified genetic disorder were excluded. Fifty participants were high-functioning persons with autism diagnosis and four had Asperger Syndrome. All had full-scale IQ >80 assessed using the Wechsler Intelligence Scale for Children, Fourth Edition (WISC-IV; Wechsler, 2003) or (for adolescents) the Wechsler Abbreviated Scale of Intelligence (WASI; Wechsler, 1999).

We enrolled 54 autistic patients, 44 males and 10 females, with a mean age of 14.5 ± 2.9 years. Twenty-seven of them were assigned to active 1.0 Hz TMS treatment (TMS group), while 27 were assigned to the WTL group. Mean age of subjects in the TMS group was 14.8 ± 3.2 years, and 14.1 ± 2.6 years in the WTL group. There was not a significant difference in either age or full-scale IQ between the TMS and WTL groups.

The study complied with all relevant national regulations and institutional policies and has been approved by the local Institutional Review Board (IRB). Participating subjects and their parents (or legal guardians) were provided with full information about the study including the purpose, requirements, responsibilities, reimbursement, risks, benefits, alternatives, and role of the local IRB. The subjects were reimbursed only for participation in two ERP tests ($25/per test). The consent and assent forms approved by the IRB were reviewed and explained to all subjects who expressed interest to participate. All questions were answered before consent signature was requested. If the individual agreed to participate, both she/he and parent/guardian signed and dated the consent or assent form and received a copy countersigned by the investigator who obtained consent.

## Three-stimuli oddball task with Kanizsa figures

**Figure 1:**
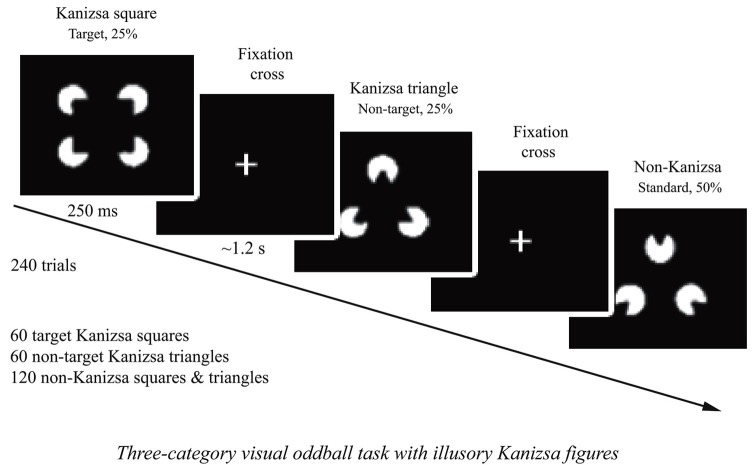
**Three-stimuli category visual oddball task with Kanizsa illusory figures.** The stimulus types are: Kanizsa square (target, 25%), Kanizsa triangle (25%), non-Kanizsa square, and non-Kanizsa triangle. The non-target Kanizsa triangle is introduced to differentiate processing of Kanizsa figures and targets. The stimuli consist of either three or four inducer disks, which are considered the shape feature, and they either constitute an illusory figure (square, triangle) or not (collinearity feature).

The stimuli employed in the test were Kanizsa square (target), Kanizsa triangle (non-target), non-Kanizsa square, and non-Kanizsa triangle (standards) (Kanizsa, 1976). The task represents a classic three-stimuli oddball with infrequent illusory Kanizsa target (square, 25%) and infrequent Kanizsa distracter (triangle, 25% ) figures presented for 250 ms among frequent non-Kanizsa stimuli (so called standards, 50%) with inter-trial interval in 1,100–1,300 ms range (Figure 1). Totally 240 trials were presented following a brief practice block. The practice block had 20 trials only with the experimenter present in the room to make sure that subject correctly understands test conditions and recognizes target stimuli. The total time of the test including sensor application and practice was under 30 min. For better habituation and adaptation to experimental setting, the participants were encouraged to have at least one session for conditioning to brainwave sensor net (without performing task) and getting familiar with laboratory environment.

## Event-related potential acquisition and processing

Electroencephalographic (EEG) signals from 128 sites were recorded with a dense-array EGI system (Electrical Geodesics, Inc., Eugene, Oregon). Subjects were placed in electrically and acoustically isolated camera from the Industrial Acoustics Co. (Bronx, NY). Stimulus presentation and MT collection was controlled using E-prime (PST, Inc., Pittsburg, PA). Visual stimuli were presented on a flat monitor located in 45–50 cm from the subject, and MTs were registered with a keypad (Serial Box, Inc). Sampling rate of EEG was 500 Hz, and analog Notch (60 Hz, infinite impulse response (IIR)) and analog elliptical bandpass filters were set at 0.1–200 Hz. Impedances were under 40 KΩ. Stimulus-locked EEG data were segmented off-line into 200 ms pre-stimulus baseline to 800 ms epoch post-stimulus. EEG recordings were screened for artifacts and trials with eye blinks, gross movements etc were removed using EGI software artifact rejection tools (Perrin et al., 1987; Fletcher et al., 1996; Srinivasan et al., 1998; Luu et al., 2001). The remaining artifact-free EEG data for trials with correct responses was digitally filtered using Notch filter (IIR, 5th order) and 0.3–20 Hz IIR elliptical bandpass filter. Averaged ERP data was baseline corrected (200 ms) and ERPs after averaging and baseline correction were re-referenced into an average reference frame. Response-locked EEGs were segmented into 500 ms pre-response to 500 ms post-response (i.e., commission error). More detailed account for experimental procedure and EEG data acquisition and processing can be found in our prior publications that used similar methodology (Baruth et al., 2010a,b; Casanova et al., 2012; Sokhadze et al., 2012a,b).

## Event-Related Potentials (ERP)

### Stimulus-locked dependent ERP variables

Dependent variables for the frontal and fronto-central region-of-interest (ROI) were N100 (80–180 ms), N200 (220–350 ms), P2a (180–320 ms), and P3a (300–600 ms), and for the parietal and parieto-occipital ROI were P100 (120–180 ms), N200 (180–320 ms) and P3b (320–600 ms) ERP waves. For P2d component (i.e., differences wave of frontal P2a) we calculated difference wave (P2a to targets minus P2a to non-targets) to detect mean difference between two conditions both in amplitude and latency within 180–320 ms post-stimulus window.

### Response-locked Event-Related Potentials (ERN/Pe)

Response locked dependent variables in this study were amplitude and latency of the Error-related Negativity (ERN peaking within 40–150 ms post-error) and Error-related Positivity (Pe, peaking within 100–300 ms post-error). The ROI for both ERN and Pe components included FCz, sites between FCz and FC3-C1, and between FCz and FC2-C2. Amplitude and latency analysis of ERN/Pe was performed with a custom-made application in Matlab (Clemans et al., 2011a). Validation of correct identification of ERN and Pe waves was further ascertained using another custom Matlab application using wavelet transformation (Clemans et al., 2011b).

## Transcranial magnetic stimulation

Repetitive TMS was administered using a Magstim 220 device (Magstim Corp., Sheffield, UK) with a 70-mm figure-eight coil. Threshold of MT was identified for each hemisphere in all participants with autism by increasing the output of the stimulator by 5% until a 50 μV deflection or a visible twitch in the First Dorsal Interosseous (FDI) muscle was detected in at least 3 trials of stimulation over the motor cortex controlling the contralateral FDI. Electromyographic (EMG ) responses were recorded with a C-2 J&J Engineering Inc multichannel physiological monitoring device with Physiodata software (J&J Engineering, Inc., Bainbridge Island, WA).

The rTMS was administered weekly for 18 weeks with the 1st six treatments were over the left DLPFC, while the next six were over the right DLPFC, whereas remaining six treatments were done bilaterally over the DLFC (evenly at the left and right DLPFC). The DLPFC site for magnetic stimulation was found by placing the TMS coil 5 cm anterior, and in a parasagital plane, to the site of maximal FDI response. A swimming cap was used to make the TMS coil positioning easier. TMS was administered at 1.0 Hz frequency and 90% MT. There were total of 180 pulses per day session with nine trains with 20 pulses each. There were 20–30 s between the train intervals used. Decision to select 90% of the MT was based on the prior publications where rTMS was used for the stimulation of DLPFC in various neuro- and psychiatric disorders (reviewed in Pascual-Leone et al., 2000; Wassermann and Lisanby, 2001; Daskalakis et al., 2002; Gershon et al., 2003; Loo and Mitchell, 2005; Greenberg, 2007; Oberman et al., 2013).

## Clinical social and behavioral evaluation outcomes

For the evaluation of social and behavioral functioning we utilized caregiver reports and clinician ratings of improvement. Every participant was evaluated before TMS course and within 2 weeks following TMS treatment. *Aberrant Behavior Checklist* (ABC; Aman and Singh, 1994; Aman, 2004) is a clinician administered rating scale to assess Irritability, Lethargy/Social Withdrawal, Stereotypy, Hyperactivity, and Inappropriate Speech based on parent/caregiver report. *Social Responsiveness Scale (SRS)*. *Repetitive Behavior Scale—Revised* (RBS-R; Bodfish et al., 1999) is a caregiver completed rating scale assessing stereotyped, self-injurious, compulsive, ritualistic, sameness, and restricted range (Bodfish et al., 2000).

## Statistical analysis

The primary model for statistical analyses of subject-averaged ERP and MT data was the two factor repeated measure ANOVA. Dependent ERP variables were amplitude and latency of ERP at pre-determined ROIs. The within-participant factors were followings: *Stimulus* (Kanizsa target, Standard, Kanizsa Non-target), *Hemisphere* (Left, Right), and *Time* (Baseline, Post-treatment). The between-subject factor was *Group* (TMS, WTL). *Post hoc* analyses were conducted where appropriate. RT, error rate (commission, omission and total error rate), were analyzed using *Time* and *Group* factor. For clinical behavioral rating scores a *Treatment* (pre-vs. post-TMS/or waiting period) ANOVA was completed to determine changes associated with active stimulation and WTL conditions. Histograms with normal distribution curves along with skewness and kurtosis data were obtained for each dependent variables to determine normality of distribution and appropriateness of data for ANOVA and *t*-tests. For more reliable determination of normality of distribution residual plots (i.e., normal probability plot, histogram, vs. fits and order) were created using Minitab statistical package to indicate that treatment with ANOVA is justified. All dependent variables in the study had normal distribution. Greenhouse-Geisser corrected *p*-values were employed where appropriate in all ANOVAs. *A priori* hypotheses were tested with the Student’s *t*-tests for two groups with equal variance. Confidence intervals (95% of mean, 95% CI) were calculated for each ERP data sets entered for *t*-tests. For the estimation of the effect size and power (Murphy and Myors, 2004) we used Partial Eta Squared (*η*^2^) and observed power computed using *α* = 0.05. SPSS 19.0 and Sigma Stat 3.1 statistical packages were used for analysis of data.

## Results

### Behavioral responses (Reaction time and accuracy, post-error RT)

#### Reaction Time (RT)

Effects of TMS on RT to targets were not significant. Comparison of RT to targets yielded no *Time* X *Group* effects.

#### Accuracy

Commission and omission errors analysis yielded a significant between-group difference in the commission error percentage, *F*_(1, 52)_ = 4.32, *p* = 0.042. *T*-test showed significant decrease of commission error rate in the TMS group (mean decrease −6.38 ± 2.54%, 95% CI from −11.61 to −1.15%, *t*_(26)_ = 2.50, *p* = 0.019). We could not find between group differences in omission error rate. Total error rate (% errors) change also showed decrease only in TMS group (−7.47 ± 2.82%, 95% CI from −13.26 to −1.67%, *t*_(26)_=2.64, *p* = 0.013).

#### Post-error RT

Main effect of *Time* (Pre, Post) on normative post-error RT slowing was highly significant (*F*_(1,50)_=15,14, *p* = 0.001, *η*^2^ = 0.134, observed power = 0.795 at alpha (α) = 0.05).

**Figure 2:**
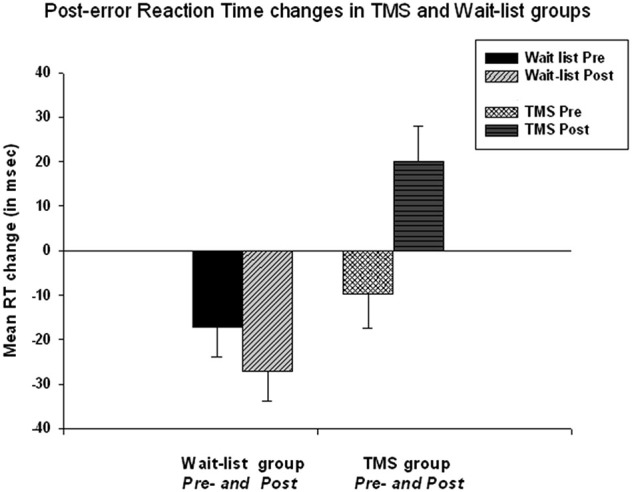
**Post-error reaction time (RT) changes in TMS and wait-list groups at the baseline and at the second test**. *Time* X *Group* effect is highly significant (*F* = 8.05, *p* = 0.006). At the baseline both groups showed post-error RT speeding, while post-TMS post-error RT became positive. Change of the post-error RT in TMS group was significant (*t* = 4.57, *p* < 0.001).

Repeated measure ANOVA of post-error RT slowing revealed that TMS and WTL group differences on post-error RT changes were also statistically significant, i.e., *Time* X *Group* interaction, *F*_(1, 52)_ = 8.05, *p* = 0.006, *η*^2^ = 0.134, observed power = 0.795. The TMS group showed post-error RT increase with significant positive change in post-error RT. This change was computed as post TMS post-error RT change minus pre-treatment post-error RT change (49.9 ± 55.4 ms, 95% CI from 26.42 to 69.41 ms, *t*_(26)_ = 4.57, *p* < 0.001). Figure 2 shows that at the baseline both in WTL and TMS groups post-error RT was negative (mean post-error speeding was −23.1 ± 34.7 ms and not different between groups at pre-treatment stage), while in the TMS group post-error RT became positive (i.e., showed normative slowing), whereas it remained negative in the WTL group.

### Parietal and parieto-occipital ERP components

#### P100

TMS course had main effects on P100 component’s both amplitude (*F*_(1,52)_ = 4.78, *p* = 0.033) and latency (*F*_(1,52)_ = 15.00, *p* = 0.001). Response of this parietal and parieto-occipital P100 component (positive peak within 130–160 ms post-stimulus) to targets showed post-treatment between group difference in amplitude (2.25 ± 2.93 μV, with 95% CI from 1.14 to 3.37 μV, in TMS vs. 4.37 ± 3.89 μV, 95% CI from 2.83 to 5.91 μV, in WTL, *F*_(1,52)_ = 5.31, *p* = 0.025) and latency (153.3 ± 43.99 ms, 95% CI from 136 to 170 ms in TMS vs. 128 ± 18.42 ms, 95% CI from 121 to 135 ms in WTL, *F*_(1,52)_ = 7.54, *p* = 0.008). Group differences in response to Kanizsa targets and non-targets were more expressed in the latency of the P100 (*F*_(1,52)_ = 4.91, *p* = 0.011). The *Stimulus* (Standard, Non-target Kanizsa, Target Kanizsa) X *Time* (Pre, Post) X *Group* (TMS, WTL) effect was significant (*F*_(2,52)_ = 4.34, *p* = 0.015), and this effect was even more powerful for standard vs. target stimuli comparison (*F*_(1,52)_ = 7.92, *p* = 0.007, *η*^2^ = 0.128, observed power = 0.789). The effect can be described as a reduced latency to non-targets and increased latency to target stimuli post-TMS but not after wait period. There were no hemispheric differences observed for P100 component.

#### N200

**Figure 3:**
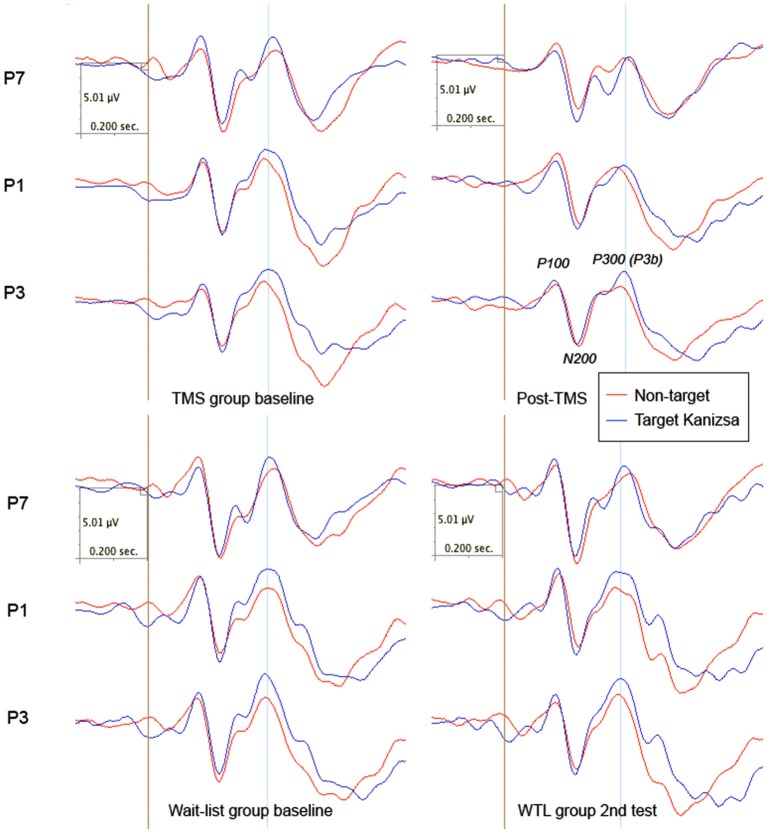
**Parietal ERPs (P1, P3, P7 sites according to 10–10 International System) to target and non-target Kanizsa figures in TMS and wait-list groups (*N* = 27/per group) before and after treatment (TMS/or wait-period)**.

There were no group differences in amplitude of the parietal N200 component. Latency of N200 to targets showed post-treatment between group difference in latency to targets (238.72 ± 58.58 ms, 95% CI from 215 to 261 ms, in TMS vs. 201.35 ± 24.27 ms, 95% CI from 191 to 210 ms, in WTL group, *F*_(1,52)_ = 9.34, *p* = 0.004) and non-target illusory Kanizsa figures (242.31 ± 62.42 ms, 95% CI from 217 to 267 ms, in TMS vs. 208.27 ± 24.92 ms, 95% CI from 198 to 218 ms, in WTL group, *F*_(1,52)_ = 6.92, *p* = 0.011). ANOVA analysis of the latency of parietal N200 to target and non-target Kanizsa stimuli showed a *Stimulus* (Target, Non-target) X *Time* (Pre, Post) X* Group* (TMS, WTL) interaction, *F*_(2,52)_ = 3.69, *p* = 0.032. The effect was expressed as increased latency for non-target stimuli in the TMS group post-treatment. There were observed other interactions as well, for instance hemispheric one, as the effect was featured by more delayed latency at the right hemisphere in the TMS group (*F*_(1,52)_ = 7.15, *p* = 0.01, *η*^2^ = 0.121, power = 0.747). Other notable interaction was significant *Time* X *Group* effect, *F*_(1,52)_ = 4.60, *p* = 0.037, *η*^2^ = 0.08, observed power = 0.558, with TMS showing more prolonged N200 latency to non-target Kanizsa stimuli. *Post hoc* tests showed that in the active treatment group latency increased (e.g., to non-targets, bilaterally 24.2 ± 11.8 ms, 95% CI from 3.7 to 51.0 ms, *t*_(26)_ = 2.37, *p* = 0.025) along with attenuated amplitude (−1.39 ± 3.26μV, 95% CI from −2.63 to −0.15 μV, *t*_(26)_ = 2.30, *p* = 0.029), while changes of latency and amplitude of N200 in the WTL group were not significant (see Figure 3).

#### P300 (P3b)

We found between group differences in P3b amplitude that were expressed as more attenuated component post-treatment in TMS as compared to WTL group only to non-Kanisza standards (2.69 ± 2.96 μV, 95% CI from 0.88 to 3.61 μV, in TMS vs. 5.09 ± 3.98 μV, 95% CI from 3.21 to 6.14 μV, in WTL, *F*_(1,52)_ = 5.25, *p* = 0.026). We found no interactions of P3b amplitude using in ANOVA *Stimulus*, *Hemisphere, Time*, and *Group* factors. *Stimulus* (Target, Non-target Kanisza, Standard) factor had a main effect on latency of P3b (*F*_(2,53)_ = 11.59, *p* < 0.001). The latency of P3b showed significant effects of *Time* factor on each stimuli: latency of P3b to targets, 364.15 ± 63.08 ms, 95% CI from 340 to 380 ms, in TMS vs. 326.13 ± 28.27 ms, 95% CI from 314 to 337 ms, in WTL, *F*_(1,52)_ = 8.16, *p* = 0.006; non-target Kanizsa, 356.68 ± 67.26 ms, 95% CI from 333 to 384 ms, in TMS vs. 322.93 ± 21.55 ms, 95% CI from 314 to 331 ms, in WTL, *F*_(1,52)_ = 6.94, *p* = 0.011; and standards, 354.89 ± 64.68 ms, 95% CI from 330 to 379 ms, in TMS vs. 323.54 ± 20.68 ms, 95% CI from 315 to 331 ms, in WTL, (*F*_(1,52)_ = 5.25, *p* = 0.026). Repeated measure ANOVA analysis of the P3b latency also indicated a significant between groups differences for all types of illusory figures, for example, increased P3b latency as a result of rTMS (*Time* × *Group* interaction, *F*_(1,52)_ = 4.32, *p* = 0.044), that can be described as a longer post-treatment latency in TMS, shorter in WTL group.

### Frontal and fronto-central ERP components

#### N100

Comparison of post-treatment amplitude and latency of N100 ERP component showed decreased amplitude and prolonged latency to both target and non-target Kanizsa figures in the TMS group, while N100 magnitude was practically unchanged in the WTL group. Effects of *Time* factor on amplitude and latency to targets was significant (at post-TMS test, amplitude, −1.54 ± 1.83 μV, 95% CI from −2.72 to −0.88 μV, in TMS vs. −2.91 ± 2.96 μV, 95% CI from −3.59 to −1.74 μV, in WTL, *F*_(1,52)_ = 4.62, *p* = 0.036; while latency, 140.85 ± 32.76 ms, 95% CI from 127 to 153 ms, in TMS vs. 120.83 ± 20.87 ms, 95% CI from 112 to 129 ms, in WTL group). Effects of *Time* on frontal N100 to non-targets was also statistically significant (amplitude, −1.36 ± 1.63 μV in TMS vs. −2.37 ± 2.07 μV in WTL, *F*_(1,52)_ = 4.47, *p* = 0.04; latency, 140.59 ± 23.22 ms in TMS vs. 125.4 ± 13.38 ms in WTL, *F*_(1,52)_ = 8.58, *p* = 0.005). There were no interaction of N100 amplitude and latency on *Stimulus*, *Hemisphere* and *Group* factors.

#### N200

**Figure 4:**
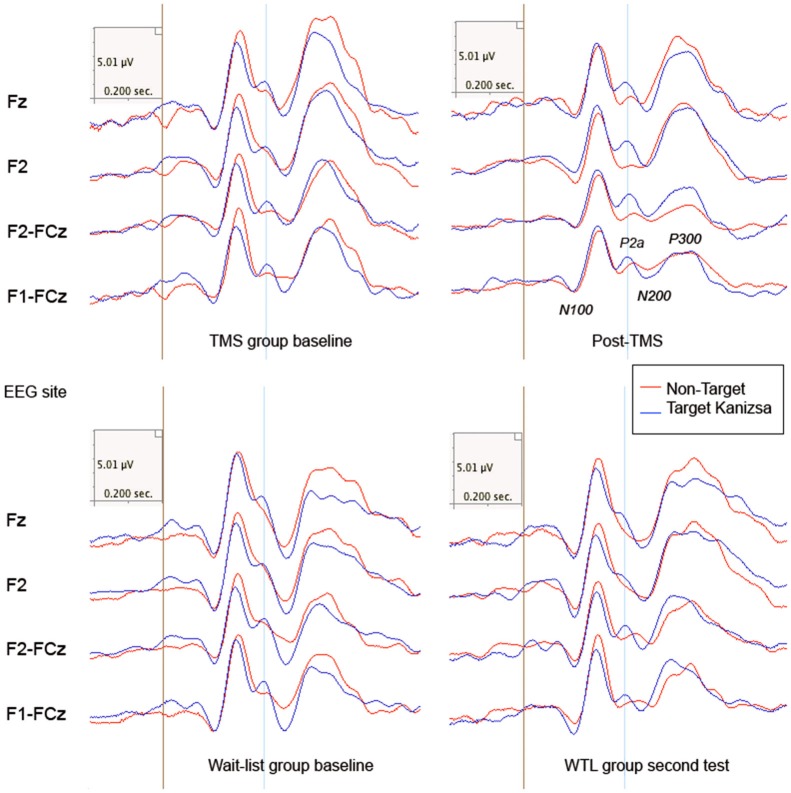
**Frontal and frono-central ERPs (Fz, F2, F2-FCz, F1-FCz) to target and non-target Kanizsa figures in TMS and wait-list groups (*N* = 27/per group) before and after treatment (TMS/ or wait-period).** Frontal P2a components (280–320 ms post stimulus) is marked with a blue line.

There was observed significant between *Group* (TMS, WTL) difference in N200 amplitude (*F*_(1,52)_ = 8.24, *p* = 0.006, *η*^2^ = 0.119, observed power = 0.804). A *Stimulus* (Target Kanizsa, Non-Kanizsa standard) X *Hemisphere* (Left, Right) X *Group* (TMS, WTL) interaction reached significance (*F*_(1,52)_ = 4.64, *p* = 0.037) pointing at a more negative N200 to targets with less negative N200 to non-target Kanizsa as a result of rTMS (Figure 4). The TMS group showed less hemispheric differences post-treatment, while the WTL group had more negative amplitude of N200 at the right hemisphere. A *Time* X *Group* effect for the latency of N200 was significant (*F*_(1,52)_ = 7.26, *p* = 0.009, *η*^2^ = 0.119, observed power = 0.754), yielding longed latency to targets post-TMS. Additionally, *post hoc* analysis using *t*-test showed that N200 latency became statistically more prolonged to target stimuli in TMS group across both hemispheres (13.31 ± 34.03 ms, 95% CI from 26.2 to 0.36 ms, *t*_(26)_ = 2.10, *p* = 0.044).

#### P2d

**Figure 5:**
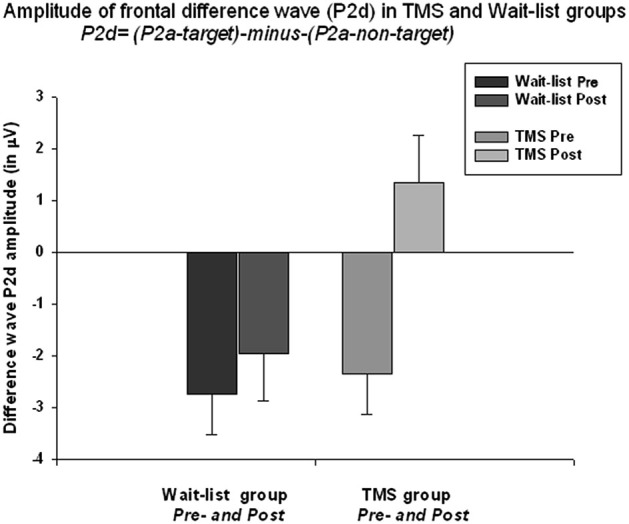
**Amplitude of the frontal P2a difference wave (so called ***P2d = [P2a to targets minus P2a to non-targets]***) across both hemispheres shows ***Time*X*Group*** interactions effect (*F* = 4.11, *p* = 0.048)**. Difference wave (P2d) was negative at the baseline in both groups (i.e., lower amplitude to targets as compared to non-targets), but becomes positive post-TMS. Increase of P2d was significant in the TMS group (*t* = 2.52, *p* = 0.018).

The frontal P2a calculated as a mean difference between P2a amplitude to target Kanizsa minus P2a amplitude to non-target Kanizsa stimuli. TMS had significant effect at P2d amplitude (*F*_(1,52)_ = 6.56, *p* = 0.013). The baseline values in both groups were similar (−2.35 μV in TMS vs. −2.51 μV in WTL) but showed significant difference post-treatment (1.34 ± 4.65 μV in TMS vs. −1.97 ± 3.56 in WTL group). ANOVA showed significant *Time* X *Group* interaction, *F*_(1,52)_ = 4.11, *p* = 0.048, *η*^2^ = 0.075, observed power = 0.512. Effect can be described as P2d becoming positive post-TMS, i.e., P2a component to targets was larger than to non-targets. Paired sample *t*-test confirmed that P2d amplitude increased significantly post-TMS (3.70 ± 7.47 μV, 95% CI from 6.71 to 0.68 μV, *t*_(26)_ = 2.52, *p* = 0.018). Differences in P2d latency between groups were not significant (Figure 5).

#### P300 (P3a)

The treatment had main effect on the amplitude of the frontal P300 (P3a) component (*F*_(1,52)_ = 4.27, *p* = 0.044). The amplitude of P3a showed a *Time* X *Group* effect that was statistically significant (*F*_(1,52)_ = 4.64, *p* = 0.036). The active TMS showed post-treatment decrease of the P3a bilaterally across all stimuli, whereas WTL group showed no differences at all. Paired sample *t*-test showed that decrease of the amplitude in TMS group was significant both for non-target Kanizsa (−1.93 ± 3.09 μV, 95% CI from −0.54 to −3.33 μV, *t*_(26)_ = 2.85, *p* = 0.008) and target Kanizsa stimuli (−2.91 ± 3.84 μV, 95% CI from −0.64 to −5.18 μV, *t*_(26)_ = 2.64, *p* = 0.014). There were not detected any main effects or interactions in the latency of the frontal P3a.

### Response-locked frontal and fronto-central ERN and Pe

**Figure 6:**
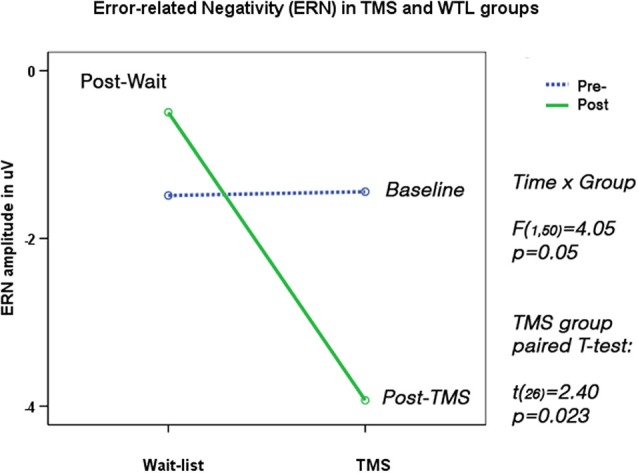
**Error-related Negativity (ERN, 40–120 ms post-error) shows *Time* X *Group* interaction (*F* = 4.05, *p* = 0.05).** Post-TMS ERN amplitude became significantly more negative (*t* = 2.40, *p* = 0.023). *N* = 26/per group, as 2 subjects out of 54 committed no commission errors on the second test.

**Figure 7:**
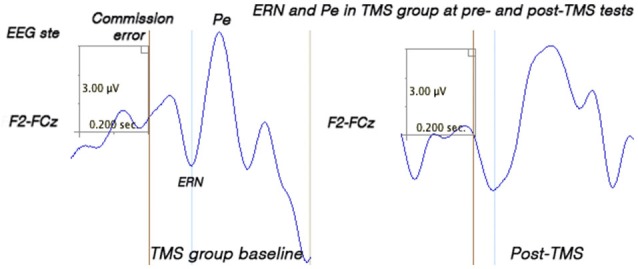
**Error-related Negativity (ERN) and Positivity (Pe) from the fronto-central midline EEG sites.** Grandaverage waveforms at the fronto-central site (between F2 and FCz, *N* = 26 per group) show more negative amplitude and shorter latency of the ERN in the TMS group post-treatment. ERN peak occurring within 40–140 ms post-error is marked by a blue line.

Two subjects did not show sufficient number of commission errors and were excluded from the analysis. TMS and WTL groups showed significant differences in ERN amplitude (*F*_(1,50)_ = 6.20, *p* = 0.016) and latency (*F*_(1,50)_ = 5.82, *p* = 0.023). Amplitude of ERN during commission errors across five frontal and fronto-central sites showed marginal *Time* X *Group* interaction (*F*_(1,50)_ = 4.05, *p* = 0.05), and paired-sample *t*-test showed significant increase of ERN negativity in the TMS group (by 2.97 ± 3.21 μV, 95% CI from 0.36 to 4.60 μV, *t*_(26)_ = 2.40, *p* = 0.023, see Figure 6). Analysis of ERN latency ANOVA yielded statistically significant *Time* X *Group* effect, (*F*_(1, 50)_ = 4.24, *p* = 0.041, *η*^2^ = 0.099, observed power = 0.55). *T*-test of the ERN latency changes in the TMS group showed significant decrease (−28.1 ± 13.8 ms, 95% CI from −4.22 to −52.1 ms, *t*_(24)_ = 2.41, *p* = 0.023). Amplitude and latency of Pe wave in both groups were not significantly changed post-treatment. Figure 7 shows ERN and Pe waveforms in two groups at the first (baseline) and at the second test.

### Clinical behavior evaluations post-TMS

**Figure 8:**
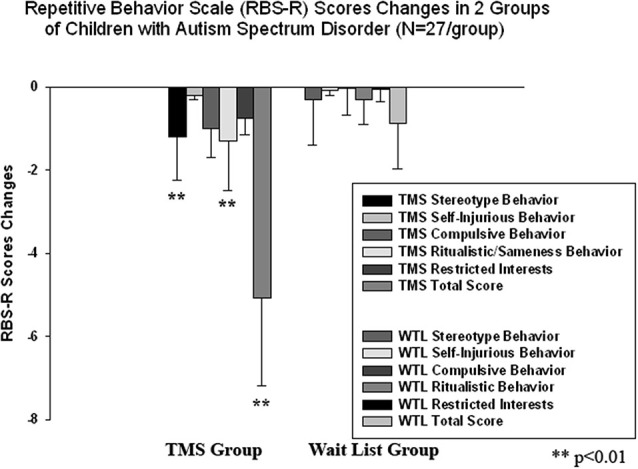
**Changes of Repetitive Behavior Scale (RBS-R) scores post-TMS/wait-list treatment as compared to baseline levels in two groups of children with ASD (*N* = 27/per group).** Stereotype Behavior, Ritualistic Behavior and Total RBS scores decreased significantly in the TMS group.

**Figure 9:**
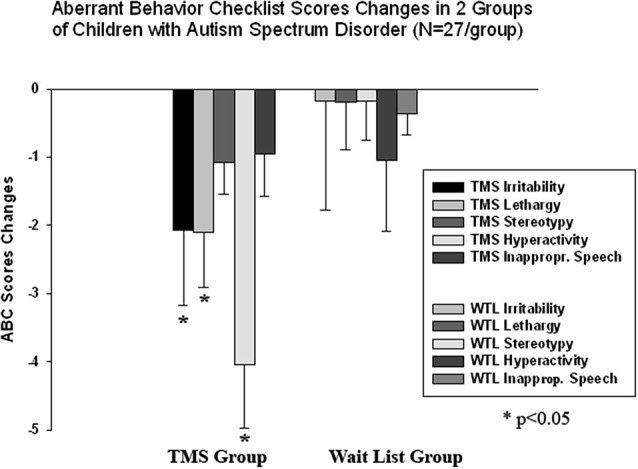
**Changes of Aberrant Behavior Checklist (ABC) scores post-TMS/wait-list treatment as compared to baseline levels in two groups of children with ASD (*N* = 27/per group).** Irritability, Lethargy, and Hyperactivity rating scores decreased significantly post-TMS.

We found a significant decrease in stereotype repetitive and restricted behavior patterns following 18 sessions of bilateral rTMS as measured by the RBS-R (Bodfish et al., 1999) and analyzed them using a paired sample Student’s *t*-test. Total RBS-R score decreased from 23.4 to 19.1, mean decrease being −4.29 ± 5.9, 95% CI from −1.95 to −6.63, *t*_(26)_ = 6.63, *p* = 0.001. Changes in individual subscale scores is depicted at the Figure 8, where both Stereotypic Behavior subscale and Ritualistic/Sameness behavior subscale scores show significant decrease (accordingly −1.00 ± 1.77, 95% CI from −0.28 to −1.71, *t*_(26)_ = 2.89, *p* = 0.008 and −1.33 ± 2.21, 95% CI from −0.45 to −2.21, *t*_(26)_ = 3.12, *p* = 0.004). There was identified as well a significant reduction in Irritability subscale as measured by the ABC (Aman and Singh, 1994), i.e., −2.07 ± 5.12, 95% CI from −0.40 to −4.10, *t*_(26)_ = 2.10, *p* = 0.045. Lethargy and Hyperactivity subscales showed even more pronounced score reductions (Lethargy, −2.11 ± 3.93, 95% CI from −0.51 to −3.72, *t*_(26)_ = 2.71, *p* = 0.012; Hyperactivity, −4.03 ± 7.68, 95% from −0.99 to −7.07, *t*_(26)_ = 2.72, *p* = 0.011). Changes of individual subscale rating scores in TMS group are depicted at the Figure 9. The WTL group had no significant differences in any of RBS-R or ABC scale ratings as a result of the waiting period.

## Discussion

Our results show significant changes in behavioral responses (accuracy, post-error RT slowing) and both early and later-stage ERP indices of task-relevant signal processing as a result of 18 sessions of low frequency rTMS treatment course in children with ASD.

Participant in TMS group showed decreased amplitude and prolonged latency of parietal P100 and N200 components to all stimuli, more for non-target cues. Parietal P3b ERP component was also prolonged without amplitude change in TMS group. In our prior study (Sokhadze et al., 2009b, 2010a, 2013b) at the parietal and parieto-occipital cortices the autism group showed significantly prolonged latency of N100 and reduced amplitude of N200 to targets as compared to neurotypical controls. Latency of the P3b was longer to distracters, without any amplitude group difference to targets and novels. The ASD group had prolonged latencies to novels but not to targets, with effect being better expressed in the right hemisphere. The results indicate the excess of efforts needed for the differentiation of targets from non-target novels in individuals with ASD. TMS treatment enhanced the process of target recognition during performance on task. Especially informative in this regard was positive change of the frontal P2d difference wave that indicates increase of P2a component to target Kanizsa stimuli vs. non-target Kanizsa stimuli, thus reflecting easier discrimination of target features of the stimuli (illusory square vs. illusory triangle).

In addition, at the same frontal topography N200 component was more negative to targets as compared to non-target illusory figures and had longer latency resulting in globally higher magnitude of N200 to targets. Following TMS course the N200 component at the frontal sites became more negative to targets, and at the same time significantly less negative to both types of non-target stimuli. The positive frontal P2a component followed by the negative ERP N200 component (both of them peaking within 280–320 post-stimulus) in visual oddball tests tasks are associated with categorization, perceptual closure and attention focusing ultimately signaling that a perceptual representation has been formed (Potts et al., 2004). This wave is enhanced if the presented stimulus contains a feature or attribute defining the target in the task according to Potts et al. (2004). It was previous reported (Sokhadze et al., 2009a; Baruth et al., 2010c) that individuals with ASD as compared to typical controls showed enhanced N200 to task irrelevant as compared to task relevant stimuli, and the finding that N200 became more negative to target Kanizsa figures and less negative to non-target distracters post-rTMS treatment indicates a trend to normalization of the response pattern pointing at an improved visual signal processing and a more effective discrimination of the target.

The results indicate a reduction of the frontal P3a both to target and non-target stimuli post-TMS in our ASD patients. In our earlier studies comparing ASD and typical controls we reported that the ASD group showed prolonged N100, P100, and P3b to targets stimuli, emphasizing a change indicative of abnormalities of sustained attention compared to controls. At the same time, the ASD group exhibited a prolonged P3a to novels, and this can be considered as a marker of impaired orientation to novelty, and ultimately decreased frontal associative and integrative functioning. In the current study our results show no differences in amplitude, though the latency of the P3a was still delayed.

Over-activation in the parietal cortex at the early stages of processing of non-targets, either standards or infrequent distracters, and at the same time under-activation of integrative frontal regions at the late stages of target processing was found to occur in autism in a similar visual task that was using three-stimuli paradigm with rare novel distracters (Sokhadze et al., 2009b, 2010a). Our results in a series of visual oddball tasks indicated enhanced and prolonged early frontal ERPs and a delayed late P3a to non-target stimuli, which would suggest low selectivity in pre-processing and at a later stage under-activation of integrative regions. Overall, this is an indication of an over-connected network where sensory inputs evoke abnormally large evoked potentials for unattended stimuli such as frequent standards and rare novel distracters at all stages of visual signal processing with signs of a reduced selectivity of the activation.

The results of the current study indicate that rTMS may have facilitated attention and target discrimination by improving conflict resolutions during processing task-relevant and task-irrelevant stimuli. The latency of posterior P3b was prolonged to targets but reduced to both non-target Kanzisa and non-Kanizsa stimuli following rTMS. The P3b has been linked to task-relevance and the decision- related character of the stimulus as it indicates memory-updating and individual trial processing closure (Picton, 1992). Earlier we (Sokhadze et al., 2009a,b, 2012b) noted that individuals with autism showed prolonged P300 peak to irrelevant distracters as compared to typical controls, which was similar to effects reported by other groups (Courchesne et al., 1989; Townsend et al., 2001). The auditory and visual sensory information processing abnormalities been described in ASD by different researchers (Kemner et al., 1994, 1999; Bomba and Pang, 2004). However, most of these studies analyzed and reported outcomes of late cognitive potentials such as centro-parietal P3b (Courchesne et al., 1989; Ciesielski et al., 1990) and frontal P3a (Townsend et al., 2001). There are only a few papers reporting short latency ERP components’ differences in individuals with autism. Majority of these studies emphasize over-activation as well as an abnormal pattern of basic perceptual processes such as low selectivity regardless of modality, abnormal top-down attentional control including delayed attentional orienting to novel stimuli, and deficits in information integration processes (Belmonte and Yurgelun-Todd, 2003). In typically developing children the fronto-central P3a occurs earlier in time as compared to parietal P300 (P3b), but in autistic subjects the P3a and P3b components were found to peak almost simultaneously over the frontal and parietal sites in a spatial attention test (Townsend et al., 2001). The latency of P3a is thought to be associated with the speed of attentional orienting to significant novel stimulus and reflects working memory processes in the prefrontal cortex. Centro-parietal P3b is usually described as a cognitive component that indexes context update and closure. This cognitive potential was found to be delayed but was not significantly attenuated in the group of children with autism as compared to typical controls (Sokhadze et al., 2009a,b, 2010a, 2012b).

The results of the study may indicate facilitation of visual target discrimination processes and enhanced habituation to task-irrelevant distracters post-TMS. We report significant improvement in the accuracy of MTs, lower total error rate and improved normative post-error RT slowing following 18 session long rTMS course. These result support our earlier findings outlining improvement in attention, executive control, and irrelevant response inhibition post-TMS treatment in autism.

In our initial rTMS pilot studies (Sokhadze et al., 2009b, 2010a) we used only six sessions of low frequency rTMS applied only to the left DLPFC and assessed behavioral performance in a visual attention task in children with autism. In a very similar manner, our current study also found a notable reduction in the frontal N200 and altered latency of the parietal P3b to task-irrelevant stimuli. Additionally, similar to the present investigation we also found a significant reduction in the response errors rate following a shorter courses of the prefrontal rTMS (Sokhadze et al., 2009b, 2012a; Baruth et al., 2010a). It might be stated that we found even more pronounced changes in cognitive ERPs such as P2d, N200and P3b in this study that had the greater number of rTMS treatments (18 sessions). In another study using this time 12 sessions of rTMS we found a significant reduction in repetitive and restricted behavior patterns as well as a significant reduction in irritability according to clinical and behavioral questionnaires (Casanova et al., 2012).The results of current 18 session-long rTMS treatment confirm and expand our prior findings of reduced repetitive behaviors (Sokhadze et al., 2009a,b, 2010a,b; Baruth et al., 2010b) and irritability (Baruth et al., 2010a,b) following low-frequency rTMS course. It should be noted that we found significant reductions in irritability only as a result of 12 sessions of bilateral stimulation (Baruth et al., 2010a), whereas reductions in repetitive behavior have been significant after six sessions of stimulation to the left DLPFC (Sokhadze et al., 2009b, 2010a).

It was a very reasonable decision to select DLPFC as a site for rTMS stimulation. The DLPFC processes components of working memory, decision making process, and regulates the ability to focus attention on task-relevant goals while inhibiting responses to distracters (Gray et al., 2003; Enriquez-Geppert et al., 2010; Matzel and Kolata, 2010). Suggested disruption in the ratio between cortical excitation and inhibition especially within the prefrontal cortex in individuals with autism (Casanova et al., 2002a, 2006a,b) was confirmed in individuals with Asperger syndrome (Casanova et al., 2002c). Reduced cortical inhibitory tone and an increased E/I ratio could adversely affect patterns of cortical activation, possibly resulting in isolated islands of coordinated excitatory activity and in a high comorbidity rate of ASD and epilepsy (Tuchman and Rapin, 1997). We believe that a course of 18 neuromodulatory sessions of low frequency rTMS may restore the cortical E/I balance by selective activation of double-bouquet cells at the periphery of cortical minicolumns (Casanova et al., 2006a,b; Casanova, 2007). It was shown that minicolumnar abnormalities in autism are most significant within the prefrontal cortex, more specifically, the DLPFC and the ACC (Fernandez-Duque et al., 2000; Mesulam, 2000; Casanova et al., 2002b, 2006a,b).

Rubenstein and Merzenich (2003) put forward a hypothesis that at some forms of autism could be caused by a disproportionate high level of excitation (E) or disproportionately weak inhibition (I) resulting in a high E/I ratio. Cortical circuits with such enhanced E/I level are proposed to be featured by poor functional differentiation which may lead to broad-ranging abnormalities in perception, memory and cognition, and motor control. Among other defects, individuals with autism have well known perceptual processing abnormalities, including a hypersensitivity to auditory, visual and tactile stimulation (Gomot et al., 2002; Plaisted et al., 2003). Studies of perceptual systems in animal models may provide useful insights into mechanisms underlying sensory disturbances in autism. In particular, investigations of auditory development in rats using modulated noise manipulation showed that the representation of sound inputs in the cortex remains poorly differentiated when the cortex is undergoing development under very poor signal-to-noise conditions (Chang and Merzenich, 2003). The E/I balance in the cortex is controlled by the relative numbers and functional activity of glutamatergic and GABA-ergic neurons. Neurodevelopmental abnormalities may lead to increased number, morphology or functional balance of excitatory vs. inhibitory neurons and can lead to a hyper-excitable state typical for autism. Excessive noise in cortical structures processing information also negatively affects development of normally differentiated representations. Relatively undifferentiated representations of orienting signals or significant stimuli would result in larger and less selective response. Such over-representation by non-differentiated responses could account for the strong aversive reactions to auditory, tactile and visual stimuli that are common in autism.

Casanova et al. (2003) study indicated that minicolumns in the brains of individuals with autism are narrow and have altered internal organization. More specifically, their minicolumns have less peripheral neuropil space, which is the conduit for inhibitory local circuit projections. A defect in these GABAergic interneurons may correlate with the increased E/I balance and prevalence of seizures among autistic patients. The authors concluded that GABAergic interneurons are vital for sensory signal processing (e.g., filtering capacity, proper signal discrimination, etc.), thus providing a putative correlate to autistic symptomatology. As it was noted in a recent review on use of TMS in ASD (Oberman et al., 2013), TMS could be particularly informative in detecting abnormalities in E/I ratios in ASD given theoretical studies regarding role of GABAergic interneurons in autism etiology (Hussman, 2001) and specifically role of high E/I balance in autism (Casanova et al., 2003; Rubenstein and Merzenich, 2003). Our current study is supportive of idea that rTMS is capable to improve E/I ratio as manifested in electrocortical responses to sensory stimulus processing in visual selective attention test.

This TMS study was guided by the “minicolumnar” theory of autism. The hierarchical basis of the modular organization of the cerebral cortex is well recognized in the literature. The cerebral cortex originates during brain development as germinal cells from the ventricular and later on the subventricular zones divide asymmetrically and the resulting neuroblasts migrate towards the pial surface (for review see Casanova and Trippe, 2006). The migrating neuroblasts split the preplate to form the incipient cortex wherein arriving cells acquire an orderly inside-out configuration by using either somal translocation or radial glia projections as a scaffold (Marín-Padilla, 1998). The resulting vertical arrangement of cells within this dynamic system serves as an attractor for satellite interneurons to populate its peripheral neuropil space. Radially migrating neurons provide for future pyramidal cells while those that follow a tangential path, primarily from the ganglionic eminences, are destined to be interneurons. Different types of interneurons form dyadic units with pyramidal cells and the resulting ensemble of cells, along with their afferent/efferent projections, constitute information processing units better known as minicolumns (Marin-Padilla, 2010). Recent studies indicate that higher cognitive functions including our executive functions derive from the workings of these modules or minicolumns (Opris et al., 2013).

Topographical studies of minicolumnar morphometry in ASD have shown the greatest deviance from neurotypicals within the prefrontal cortex (Casanova et al., 2002d, 2006a, 2010). Some investigators have explained this fact as resulting from the prolonged maturation time of this structure which thus provides a larger time window of opportunity for exogenous factors to alter its development (Opris and Casanova, 2014). Within the rostral brain region abnormalities within the DLPFC could serve as a pathological correlate to observed executive function deficits in autism (Opris and Casanova, 2014). Given the vertical orientation of inhibitory elements within the periphery of the minicolumns (e.g., double bouquet cells) it has been proposed that rTMS in ASD could preferentially help build the inhibitory surround of these modular structures. Since the dorsolateral prefrontal cortex has been a source of significant minicolumnopathy in published postmortem studies it could be viewed as a target for stimulation using rTMS (Casanova et al., 2002b, 2012). Furthermore, considering the trans-synaptic effects of rTMS, the large number of DLPFC connections could provide a therapeutic cascading effect in other parts of the brain. In autism computerized image analysis suggests the presence of a minicolumnopathy characterized by an increased density of modules and a diminution in their peripheral neuropil space (Casanova et al., 2002a). The deficits previously described by our group have been corroborated using a variety of neuronomorphometric techniques (e.g., Euclidean minimum spanning tree, gray level index), in an independent sample conducted by an international study where the investigators were blind to the study variables, and in the published results of other investigators (Casanova et al., 2002d, 2006a; Buxhoeveden et al., 2006). The diminished width of the minicolumnar peripheral neuropil space is seen throughout laminae II-VI, suggesting a deficit of an anatomical element in-common to all layers (Casanova et al., 2010). Since inhibitory elements populate all layers of the lateral compartment of the minicolumn pathology involving these elements could contribute to a deficit in the lateral or peripheral inhibitory surround of these modules. These findings gain credence from EEG recordings using lateral masking paradigms and threshold studies using flutter stimuli that sustain the presence of a lateral inhibitory deficit in autism (Kéïta et al., 2011; Puts et al., 2014). It is plausible to propose that low frequency rTMS is increasing inhibitory tone and improving lateral inhibition, and this may result in an enhancement of executive functions.

Executive function deficits were always in the center of attention in autism research. Executive function of behavioral performance monitoring comprises error detection and response conflict monitoring, functions that can be measured using response-locked ERPs such as ERN and Pe (Gehring et al., 1993; Carter et al., 1998; Van Veen and Carter, 2002; Mars et al., 2005; Arbel and Donchin, 2009, 2011). The ERN is a well-studied component whose parameters were investigated under different experimental task conditions, and its ties to error processing have been well established (Carter et al., 1998; Falkenstein et al., 2000; Gehring and Knight, 2000; Van Veen and Carter, 2002). There is an increased number of research studies examining ERN during commission errors in children (Davies et al., 2004). It is established that executive functions normally improve with age (Huizinga et al., 2006) along with demonstration that the ACC, which is now associated with executive performance monitoring, undergoes important maturation changes from childhood into adolescence, and then into adulthood (Arbel and Donchin, 2009, 2011). Furthermore, the studying error processing maturation can be used to understand mechanisms of various neurodevelopmental disorders, such as ADHD and ASD, which feature impairments in execute control (Liotti et al., 2005; Vlamings et al., 2008; Zhang et al., 2009; Sokhadze et al., 2010b). The ERN abnormalities are interpreted as reflecting early error processing impairments. A number of studies have investigated the functional relationship between the ERN and the fronto-central stimulus-locked N200, while some suggest that they represent distinct neurophysiological processes (Ridderinkhof et al., 2004), others suggest they represent different time points of the same process of response conflict monitoring (Yeung and Cohen, 2006).

One of the most important findings of current study was replication of the increase of ERN amplitude and shortened latency post-TMS reported in previous study using 12 sessions of rTMS (Sokhadze et al., 2012a). In accord with our previous study (Sokhadze et al., 2012a), the Pe component did not change post-TMS. This component has a more posterior topography and is expressed as a positivity elicited after the ERN (Falkenstein et al., 2000; Nieuwenhuis et al., 2001; Overbeek et al., 2005). In our earlier study with rTMS application in ASD (Sokhadze et al., 2009a,b, 2010a; Baruth et al., 2010c) we found that most of EEG changes such as ERP and evoked EEG gamma frequency oscillations occurred at the early stages of visual stimulus processing (e.g., less than 200 ms post-stimulus), and resulted in a better discrimination of target from non-target stimuli. Facilitation of target recognition following TMS treatment and more effective early inhibition of non-target distracters leads to less pronounced carryover of non-target over-processing. We suggested earlier that more expressed positive neuromodulation effects in the early ERPs rather than in the late ERPs might be due to enhanced suppression of task- irrelevant stimuli and less effortful discrimination of targets from non-targets during attention task performance.

One more critical methodological issue to be considered in absence of significant TMS effects on Pe in autism might be related to the number of commission errors as this measure depends on the actual number of committed errors (Franken et al., 2007). It is feasible to suggest that the magnitude of the Pe was affected by the reduced number of commission errors in active TMS group. Our prior investigation of ERN/Pe complex in autism (Sokhadze et al., 2010b) also did show Pe differences between ASD and typical children on the similar visual oddball task, but these differences were found only in a form of a significantly prolonged latency of the Pe in ASD group. There is a possibility of a dissociation of ERN and Pe effects since generation of Pe wave might be affected by the absence of feedback about the accuracy of the MR resulting in that lower awareness of error (Hewig et al., 2011).

In general, our findings are in concordance with a recent review of rTMS applications in autism research and treatment (Oberman et al., 2010, 2013). In that review the authors concluded that, though results of published studies are promising suggesting that specific rTMS protocols (Enticott et al., 2010, 2012, 2013; Fecteau et al., 2011) targeting selected regions of cortex may lead to improvement in behavioral deficits in some individuals with ASD, the therapeutic results have been still of preliminary character and additionally, the large-scale, controlled trials necessary to establish the safety and efficacy these neuromodulation protocols have to be conducted (Oberman et al., 2010, 2013).

Some limitations to the study should be taken into account. It is often reported in rTMS studies that effects of magnetic stimulation usually do not wash out in approximately one week. We believe that switching to once per week session regimen, (e.g., Casanova et al., 2012; Sokhadze et al., 2012a) improved our protocol and resulted in better clinical outcome measures. Probably the length of staying in the rTMS treatment rather than intensity is one of the main keys of behavioral and electrocrtical improvements that we observe in our later rTMS trials in ASD (Baruth et al., 2010b, 2011; Sokhadze et al., 2010a, 2012a; Casanova et al., 2012). It should be recognized that the power (90%) and schedule (number of magnetic pulses delivered per each session, 10–20 s break between trains, etc.) of our rTMS is relatively lower than those used by other TMS treatment protocols. However, it must be mentioned that other known TMS protocols were targeting psychopathologies such as treatment-resistant major depression, or neurological disorders such as for instance Parkinson disease in adults. One more limitation of the study is the use a waiting-list group as a control group rather than using a randomized clinical trial (RCT) design with a sham rTMS condition. Even though our group has a custom-made sham Magstim TMS coil and interface enabling blinding of TMS delivery, we considered this study as a preliminary pilot with a WTL group design, and plan to consider progression to a RCT design on the future stages. It is possible to consider as a limitation also the difficulty of proving in non-invasive human brain research that low frequency rTMS is activating primarily double-bouquet inter-neurons. We hope that future neurophysiological studies on animal models would be able to find support for our hypothesis.

In conclusion, the study showed that treatment with “slow” rTMS improved ERP indices of attention to targets, reduced over-reactivity to non-targets, significantly reduced MT errors to target stimuli, and enhanced response-locked potentials reflective of error monitoring and correction (e.g., ERN to commission errors, post-error RT slowing, etc). We also found significant reductions in both repetitive and stereotypic behaviors, reduced repetitive behaviors, hyperactivity and irritability scores according to social and behavioral clinical evaluations post-TMS. We consider that it is possible to conclude that neuromodulation using low frequency, inhibitory rTMS improved executive functioning and behavior in autism. This study provides further support to the statement that TMS can be regarded as a perspective treatment targeting core symptoms of ASD such as executive function deficits.

## Conflict of interest statement

The authors declare that the research was conducted in the absence of any commercial or financial relationships that could be construed as a potential conflict of interest.
